# Differential Cell Lysis Among Periodontal Strains of JP2 and Non-JP2 Genotype of *Aggregatibacter actinomycetemcomitans* Serotype B Is Not Reflected in Dissimilar Expression and Production of Leukotoxin

**DOI:** 10.3390/pathogens8040211

**Published:** 2019-10-30

**Authors:** Anne Birkeholm Jensen, Marianne Lund, Niels Nørskov-Lauritsen, Anders Johansson, Rolf Claesson, Jesper Reinholdt, Dorte Haubek

**Affiliations:** 1 Section for Pediatric Dentistry, Department of Dentistry and Oral Health, Health, Aarhus University, 8000 Aarhus, Denmark; abj@dent.au.dk; 2 Department of Clinical Microbiology, Aarhus University Hospital Skejby, 8200 Aarhus, Denmark; marialun@rm.dk (M.L.); nielnoer@rm.dk (N.N.-L.); 3 Section of Molecular Periodontology, Department of Odontology, Faculty of Medicine, Umeå University, 901 87 Umeå, Sweden; anders.p.johansson@umu.se; 4 Division of Oral Microbiology, Department of Odontology, Faculty of Medicine, Umeå University, 901 87 Umeå, Sweden; rolf.claesson@umu.se; 5 Department of Biomedicine, Aarhus University, 8000 Aarhus, Denmark; reinholdt8240@gmail.com

**Keywords:** mRNA assay, quantitative ELISA, cell lysis assay, leukotoxin, JP2 genotype

## Abstract

Leukotoxic potential of *Aggregatibacter actinomycetemcomitans* strains has been studied by the use of several methods, and results differ depending on the methods used. The aim of the present study was to perform a comprehensive examination of the leukotoxic potential of a collection of *A. actinomycetemcomitans* strains by use of three quantitative methods, Western blotting, ELISA, and mRNA expression assay and compare these results with previous data obtained by a cell lysis assay. A higher leukotoxic potential among JP2 genotype strains compared to non-JP2 genotype strains of *A. actinomycetemcomitans* was found by Western blotting, ELISA and mRNA expression assay. Leukotoxicity as determined by cell lysis assay showed a variation among strains examined, not only depending on being part of JP2 genotype *vs*. non-JP2 genotype group of *A. actinomycetemcomitans*. The leukotoxicity of *A. actinomycetemcomitans* strains as determined by cell lysis assay did not correspond to the leukotoxic potential of *A. actinomycetemcomitans* strains as determined by three quantitative methods. A comparison of the results obtained by ELISA and mRNA expression assay showed a reasonable correlation between these two methods. It seems important to use more than one method to assess the LtxA-related virulence capacity of *A. actinomycetemcomitans* in order to obtain comprehensive understanding of the leukotoxic potential of *A. actinomycetemcomitans* strains.

## 1. Introduction

*Aggregatibacter actinomycetemcomitans* is a Gram-negative member of the human oral microbiota [1] and is involved in human infections and diseases [2,3,4,5]. One of these diseases is periodontitis, characterized by destruction of tooth-supporting periodontal tissues due to an inflammatory response [6]. *A. actinomycetemcomitans* possesses several virulence factors [3], of which the production of the RTX (repeats-in-toxin) leukotoxin (LtxA) has received much attention [7,8,9,10,11,12]. LtxA induces cell lysis, degranulation and an inflammatory response in human leukocytes by interaction with the β2-integrins in the cell membrane of human immune cells [8,13,14]. A 530-bp deletion in the promoter region of the LtxA gene operon was identified in the original JP2 strain, cultured from a young individual diagnosed with juvenile periodontitis [15]. This discovery marked the beginning of an era, where the detection of the 530-bp deletion categorized highly leukotoxic *A. actinomycetemcomitans* strains as a member of the JP2 clone of *A. actinomycetemcomitans*, possessing enhanced virulence. Based on in vitro studies, it was reported that the JP2 genotype of *A. actinomycetemcomitans* has a 10–20 fold higher lytic activity than the non-JP2 genotypes of *A. actinomycetemcomitans* [15]. Furthermore, longitudinal clinical studies reported a clear correlation between being carrier of the JP2 genotype of *A. actinomycetemcomitans* and having an increased risk of developing periodontitis at a young age [16,17].

Often the leukotoxic potential of *A. actinomycetemcomitans* is characterized by the leukotoxicity in in vitro studies [11,15,18,19,20,21]. This leukotoxicity is determined by use of cell lysis assays, where human immune cells are exposed either to the bacteria or to purified LtxA, and most often the leukotoxicity is correlated with the JP2 or non-JP2 genotype of the *A. actinomycetemcomitans* strain. Therefore, it has been the common believe that the leukotoxicity of an *A. actinomycetemcomitans* strain could be explained by the 530-bp deletion, an explanation supported by recent results presented by Sampathkumar and co-workers [21]. However, *A. actinomycetemcomitans* strains without the 530-bp deletion with high leukotoxicity according to results obtained by cell lysis assay have been reported on [20]. Furthermore, the leukotoxic potential of *A. actinomycetemcomitans* strains has been explained by different mechanisms [15,18,19]. A higher expression of the mRNA, encoding the *ltxA,* has been reported to correspond with a higher leukotoxic potential [15,18], but also a variation in the activity of LtxA in some *A. actinomycetemcomitans* has been reported [19]. Only a few studies have compared the leukotoxicity of *A. actinomycetemcomitans* with the LtxA expression and production defined as a quantification of the LtxA, e.g., by enzyme-linked immunosorbent assay (ELISA), or mRNA coding for LtxA [15,18,21], but it seems reasonable to assume that the two factors are related.

In the present study, we aimed to investigate if the leukotoxicity of a collection of Ghanaian *A. actinomycetemcomitans*, serotype b, is related to the LtxA production of the strains determined by Western blotting and ELISA, and LtxA expression as determined in a mRNA expression assay. Furthermore, we wanted to compare the results obtained by ELISA and mRNA expression assay to elucidate the relationship between the two quantification methods used to determine leukotoxic potential as the LtxA production and expression of *A. actinomycetemcomitans*, respectively.

## 2. Results

### 2.1. LtxA Expression and Production of the 20 Ghanaian Strains by Western Blotting, ELISA and mRNA Expression Assay

Western blotting clearly showed that the JP2 genotype strains of *A. actinomycetemcomitans* had a higher LtxA production than the non-JP2 genotype strains (Figure S1).

The non-JP2 genotype strains 575G, 605G, 638G, 443G, and 486G were previously characterized as having high leukotoxicity by cell lysis assay (Table 1) [20].

Strains 443G and 486G might have a tendency towards a higher LtxA production than the other non-JP2 genotype strains, but not at comparable levels as to the JP2 genotype strains. Furthermore, the Western blotting demonstrated that the strains with high LtxA production had a higher amount of LtxA in the growth supernatant than in the cell pellet extract (Figure S1).

**Figure 1:**
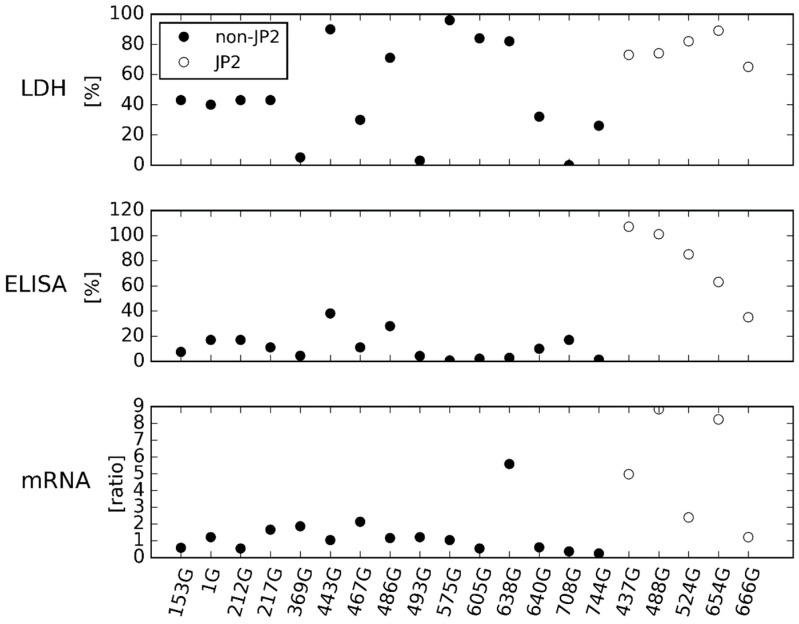
Leukotoxicity, leukotoxin production and leukotoxin expression of 20 Ghanaian *A. actinomycetemcomitans*, serotype b, JP2 and non-JP2 genotype strains determined by a cell lysis assay (LDH), an ELISA, and a mRNA expression assay.

**Figure 2:**
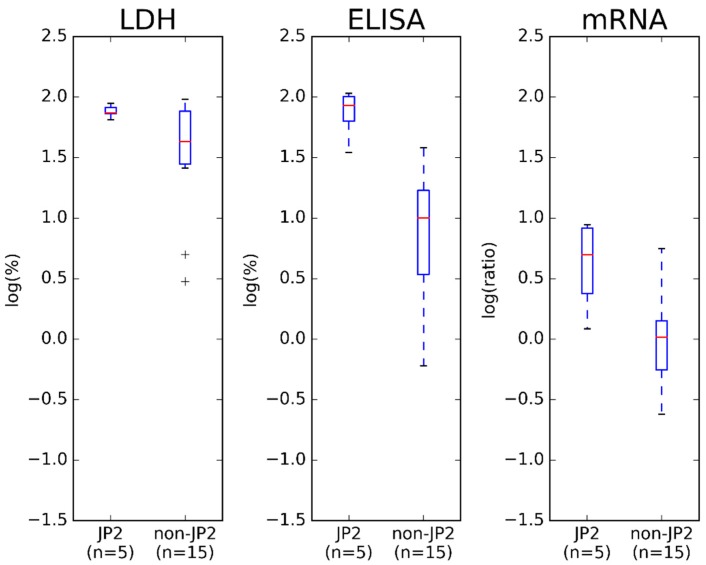
Box plot of the distribution of the 20 Ghanaian *A. actinomycetemcomitans* strains according to JP2 genotype and non-JP2 genotype strains.

By visual inspection of Figure 1, the ELISA showed the same division of JP2 and non-JP2 genotype strains of *A. actinomycetemcomitans* as demonstrated by the Western blotting (Figure S1). In Figure 1 and Figure 2, the cell lysis assay (LDH) characterizes the leukotoxicity of the strains, and the results are from the publication by Höglund Åberg and coworkers (2014) [20]. The results are given as a percentage of total lysis of THP-1 cells by Triton X. The ELISA determines the leukotoxic production of the *A. actinomycetemcomitans* strains as a percentage of the reference *A. actinomycetemcomitans* strain HK921, and results are the mean of two separate runs. The mRNA expression assay determines the leukotoxin expression of *A. actinomycetemcomitans* strains as a ratio to *adk* and *pgi*, and the results are the mean of three separate runs.

Two non-JP2 genotype strains of *A. actinomycetemcomitans* (443G and 486G) showed almost the same LtxA production as one of the JP2 genotype strains of *A. actinomycetemcomitans*. However, in general the JP2 genotype strains of *A. actinomycetemcomitans* showed a higher LtxA production than the non-JP2 genotype strains of *A. actinomycetemcomitans*. Furthermore, this finding was supported by comparing the group of JP2 genotype strains of *A. actinomycetemcomitans* to the group of non-JP2 genotype strains of *A. actinomycetemcomitans* (Figure 2). The group of JP2 genotype of *A. actinomycetemcomitans* had a statistically significant higher LtxA production than the group of non-JP2 strains of *A. actinomycetemcomitans* (*p* < 0.05) (Figure 2).

Expression of mRNA encoding LtxA did not correlate with the leukotoxicity either (Figure 1). By visual inspection, the mRNA expression assay showed a greater variation in the LtxA expression of the *A. actinomycetemcomitans* strains and found one non-JP2 genotype strain (638G) with a LtxA expression at comparable levels to the JP2 genotype strains. The average mRNA expression of JP2 genotype strains of *A. actinomycetemcomitans* was higher than observed for non-JP2 genotype strains, and the difference did attain statistical significance (Figure 2) (*p* < 0.05).

Conclusively, the high leukotoxicity of some of the non-JP2 strains of *A. actinomycetemcomitans* found by Höglund Åberg and coworkers (2014) could not be reproduced according to the LtxA expression and production found by neither the Western blotting, the ELISA, nor the mRNA expression assay.

### 2.2. Comparison of Results Obtained by ELISA and mRNA Expression Assay

**Figure 3:**
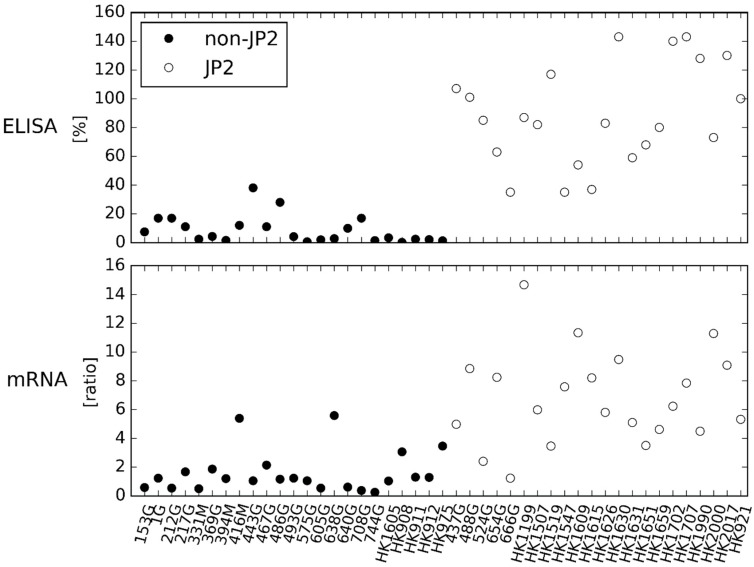
The distribution of the expanded bacterial collection of 45 *A. actinomycetemcomitans* strains for comparison of the results obtained by ELISA and in mRNA expression assay.

For further comparison of the two quantitative methods, ELISA and mRNA expression assay, 45 strains of *A. actinomycetemcomitans*, serotype b, were analyzed (Figure 3). The ELISA determines the leukotoxin production of the *A. actinomycetemcomitans* strains as a percentage of the reference strain HK921, and the results are the mean of two separate runs. The mRNA expression assay determines the leukotoxin expression of the *A. actinomycetemcomitans* strains as a ratio to *adk* and *pgi*, and the results are the mean of three separate runs.

**Figure 4:**
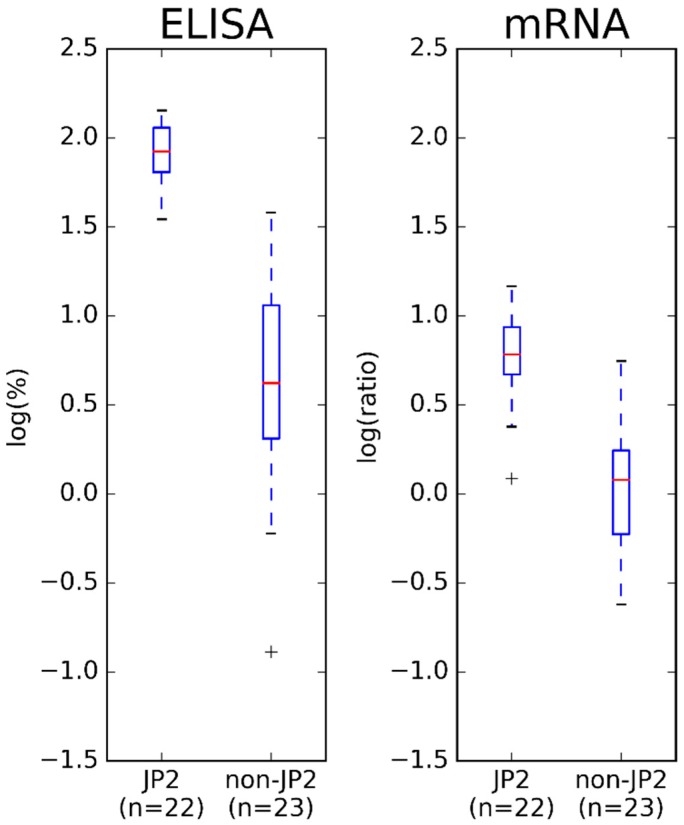
A comparison of the group of JP2 genotype strains and the group of non-JP2 genotype strains of *A. actinomycetemcomitans* based on the expanded bacterial collection of 45 strains for comparison of results obtained by ELISA and in mRNA expression assays by use of log-transformed data.

By visual inspection of Figure 3, both methods divided the *A. actinomycetemcomitans* strains, serotype b, according to their genotype, although the division was most clearly demonstrated by ELISA. A comparison of the group of JP2 genotype strains of *A. actinomycetemcomitans* and the group of non-JP2 genotype strains of *A. actinomycetemcomitans* is illustrated in Figure 4.

As for the analysis of the 20 Ghanaian *A. actinomycetemcomitans* strains, the group of JP2 genotype strains had a higher LtxA production and expression than the group of non-JP2 genotype strains of *A. actinomycetemcomitans* (*p* < 0.05) by both ELISA and mRNA expression assay.

**Figure 5:**
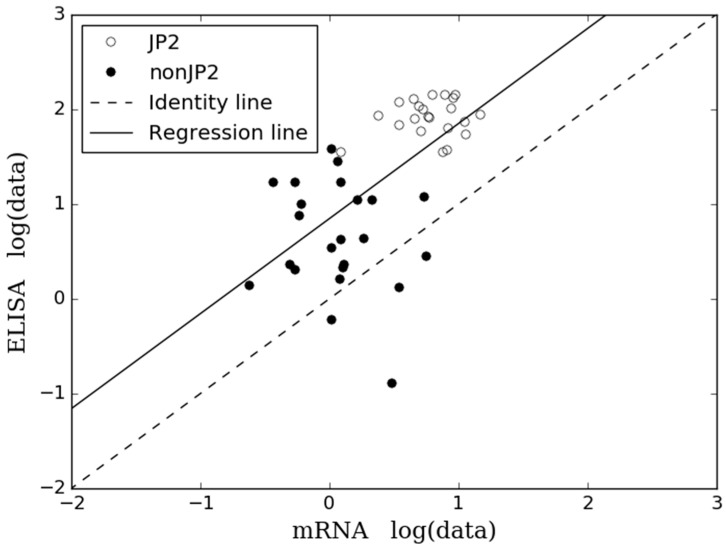
A comparison of ELISA and mRNA expression assay by an ordinary least square regression model based on the expanded bacterial collection consisting of 23 non-JP2 genotype strains of *A. actinomycetemcomitans* (black dots) and 22 JP2 genotype strains of *A. actinomycetemcomitans* (white dots) by use of log-transformed data.

By visual inspection of the scatter plot (Figure 5), where the points correspond to a particular strain, a reasonable fit between the results obtained by the ELISA and in the mRNA expression assay was seen.

However, the points tended to fall above the identity line indicating bias in the data. An ordinary least squares regression model was used to compare the ELISA and the mRNA expression assay based on the results of the collection of 45 *A. actinomycetemcomitans*, serotype b, strains. The regression fit of the two methods is given by the R^2^ at 0.37 indicating a reasonable relationship between the methods. The intercept differed from 0.0 (0.85) indicating that the regression model possesses consistent bias. A slope > 0.00 (1.00) (*p* < 0.05) illustrated a proportional (log-log linear) relationship between the two methods. Furthermore, the scatter plot (Figure 5) illustrated a greater variation in the data among the non-JP2 genotype of *A. actinomycetemcomitans*, whereas the data for the JP2 genotype strains of *A. actinomycetemcomitans* was more equal.

## 3. Discussion

In the present study, we aimed to investigate if the leukotoxic potential determined by a leukotoxicity assay of 20 Ghanaian *A. actinomycetemcomitans*, serotype b, is related to the LtxA production and expression of the strains determined by three different quantitative methods. Quantification of the LtxA production and expression using Western blotting, ELISA and mRNA expression assay could not reproduce the high leukotoxicity found among some of the Ghanaian non-JP2 genotype strains by use of cell lysis assay previously reported on by Höglund Åberg and coworkers (2014) [20]. All JP2 genotype strains of *A. actinomycetemcomitans* showed high LtxA expression and production. Furthermore, the non-JP2 genotype strains of *A. actinomycetemcomitans*, serotype b, previously characterized as having a high leukotoxicity, were all below the JP2 genotype strains when using LtxA expression and production assays. Furthermore, supplementary analysis of the expanded bacterial collection of *A. actinomycetemcomitans* strains, serotype b, revealed a reasonable relationship between the results obtained by ELISA and mRNA expression assay.

To our knowledge, this is the first study relating the leukotoxicity of a large collection of *A. actinomycetemcomitans* strains, serotype b, to the LtxA expression and production determined by three different quantitative methods. Studies have previously related the leukotoxicity of *A. actinomycetemcomitans* strains to the LtxA expression and production, often determined by quantification of mRNA or by Western blotting [15,18,21]. In addition, many studies have related a high leukotoxicity and a high LtxA expression to the presence of the 530-bp deletion in the strains [14,15,18,21,22]. The results from the present study do agree with such previous findings (Figure S1, Figure 1, Figure 2, Figure 3, Figure 4 and Figure 5). Both ELISA and Western blotting characterize the JP2 genotype of *A. actinomycetemcomitans* as having a higher LtxA production than the non-JP2 genotype strains (Figure S1 and Figure 1). Furthermore, the comparison of the group of JP2 genotype strains and the group of non-JP2 genotype strains did classify the JP2 genotype strains with a higher LtxA expression and production than the non-JP2 genotype strains regardless of using mRNA expression assay or ELISA (Figure 2 and Figure 4). The difference between JP2 and non-JP2 genotype strains of *A. actinomycetemcomitans* applied to both the collection of the 20 Ghanaian *A. actinomycetemcomitans* and to the expanded collection consisting of 45 *A. actinomycetemcomitans*, serotype b. Surprisingly, a poor relation between the leukotoxicity and the LtxA expression and production of the Ghanaian *A. actinomycetemcomitans* strains, serotype b, was found (Figure S1 and Figure 1).

Since other researchers have reported on correlating data concerning the leukotoxicity and the LtxA expression, e.g., the mRNA expression, of *A. actinomycetemcomitans,* one has to speculate if methodological complexities may explain the lack of correlation between the results obtained by cell lysis assay compared to the three quantitative assays in the present study. It has been proposed that other factors than the *ltxA* expression, LtxA production, and LtxA secretion may influence the leukotoxic potential of a strain; Diaz and co-workers did describe a higher activity of the LtxA in a fresh clinical strain compared to the original JP2 strain [19]. Still, this higher activity of the protein did correlate to a higher amount of LtxA detected on the cell pellet of the clinical strain analyzed by Western blotting [19]. Diaz and co-workers also reported that the LtxA from the fresh clinical *A. actinomycetemcomitans* strain of JP2 genotype was located on the cell pellet in larger amounts than in the supernatant [19]. However, the clinical strain was described with features comparable to a rough strain phenotype. Kachlany and co-workers studied the difference between rough and smooth strains of *A. actinomycetemcomitans*, and they found that the smooth strains secreted more LtxA into the growth supernatant than the rough strains [23]. In order to accommodate the risk of differences between the strains due to rough-smooth phenotype in the present study, all strains were converted to smooth phenotypes, and the procedures of each method were adjusted according to this. Also in the study by Åberg and coworkers, analyzing the leukotoxicity of the Ghanaian *A. actinomycetemcomitans*, serotype b, smooth strain variants were used [20]. Still, the LtxA was purified from the cell pellet and not from the growth supernatant. However, when using the Peptone-Yeast-Glucose (PYG) medium (as done in the study by Åberg and co-workers) LtxA stays attached to the cell membrane and is not secreted into the growth medium [10]. The LtxA was extracted by NaCl in the study by Åberg et al. 2014; a protocol that has been criticized by others [19]. In addition, the use of different culture broth could also interfere with the LtxA production. However, whether or not these methodological aspects may explain the lack of correlation between the leukotoxicity and the LtxA expression and production among the Ghanaian *A. actinomycetemcomitans*, serotype b, found in this study, has to be a matter of further investigation in the future.

Environmental factors are of great importance and have been reported to influence on both the production and the secretion of the LtxA [18,22,23,24,25]. Previous studies have reported that the secretion of LtxA occurs into the environment both as a water-soluble protein and as an attached protein to membrane vesicles [26,27,28]. The LtxA attached to the vesicle is expected to be found in the supernatant used in the ELISA and the Western blot; however, this is not the case for the cell lysis assay used by Åberg and coworkers [20]. Although, the amount of cell membrane-attached LtxA and the vesicle membrane-attached LtxA should be relative [29], Kato et al. showed a 5-fold greater leukotoxic activity in the LtxA purified from the vesicles compared to the cell membrane-attached LtxA [29]. Therefore, the aspect of membrane vesicles may contribute to the surprising results in the present study as well.

The results from the present study show that the leukotoxicity of the Ghanaian *A. actinomycetemcomitans* strains is not explained by a greater leukotoxin expression and production. Johansson and coworkers have described different genetic similarities among the highly leukotoxic strains in the Ghanaian collection of *A. actinomycetemcomitans* [30]. The genetic similarity among the strains characterized by the *cagE* may be an indicator of a higher toxicity of the strains, but perhaps not an indicator of a higher leukotoxicity. The protocol used for purification of the LtxA in the study by Åberg and coworkers [20] may lead to presence of other protein and membrane components in the supernatant added to the THP-1 cells used in the cell lysis assay to measure the leukotoxicity of the *A. actinomycetemcomitans* strains [31]. Therefore, it is possible that the activity of the Ghanaian *A. actinomycetemcomitans* strains towards THP-1 cells is influenced by other components, either by interference directly with the THP-1 cells or by interference with the activity of the LtxA. It would be interesting to reproduce the leukotoxicity of the Ghanaian *A. actinomycetemcomitans* strains with other LtxA-purification protocols, where the LtxA is more strictly purified [11,19,32].

The leukotoxicity reported by Åberg and co-workers is given as a percentage of total lysis of the THP-1 cells by Triton X [20]. The results of the three LtxA-quantitative methods (Western blotting, ELISA and mRNA expression assay) used in the present study do not have a maximum value in the same way, and therefore it is possible for a strain to be characterized with a value above, e.g., 100%. If a certain amount of LtxA is needed for the total lysis of THP-1 cells [18], it is possible for strains with different amounts of produced and secreted LtxA to be classified with the same level of high leukotoxicity. However, they may still be very different according to LtxA expression and production. Therefore, some of the Ghanaian non-JP2 genotype strains of *A. actinomycetemcomitans,* serotype b, might produce enough LtxA to lead to a high percentage of cell lysis, but still not produce comparable amounts of LtxA to the JP2 genotype strains of *A. actinomycetemcomitans*. Of course, such presumptions depend on the fact that there is no difference in the activity of the LtxA. This is a notion that is unanswered for the bacterial collections used in the present study.

The results obtained by use of the ELISA and the mRNA expression assay did not correlate completely. Still, there is a reasonable relationship between the methods by visual inspection of Figure 3 and Figure 5. Furthermore, both methods did determine the group of JP2 genotype of *A. actinomycetemcomitans*, serotype b, with a statistical significant higher leukotoxin expression and production than the group of non-JP2 genotype strains (Figure 4). The ELISA and the mRNA expression assay are both quantitative assays, and comparison of the results by a regression model seems reasonable [33,34]. Therefore, we performed a Bland-Altman plot for detection of difference between methods (not shown) [34], but this analysis showed no bias in our data; a result refuted by our scatter plot analysis. However, Ludbrook (2002) does discuss that the Bland-Altman analysis is not always safe to use when comparing methods that do not measure the exact same quantity and have a proportional relationship [33]. The results in the present study did show a reasonable relationship (Figure 5) when studying the scatter plot and the regression model, but with a higher agreement between the two methods among the JP2 genotype strains of *A. actinomycetemcomitans* than for the non-JP2 genotype strains. Generally, the data based on analysis of JP2 genotype strains seems more equal than for the non-JP2 genotype strains. This is probably reasonable considering that the group of JP2 genotype strains of *A. actinomycetemcomitans* is a very genetically homogeneous group of isolates, whereas the non-JP2 genotype strains, although all being of serotype b, is a more genetically diverse group of isolates. Still, based on the visual inspection of the Figure 3 and Figure 5, it seems reasonable to assume that one can predict the LtxA production based on an analysis by use of a mRNA expression assay.

Some limitations of the present study should be addressed. Our mRNA analysis consists of quite high standard deviations, but our results from the mRNA expression assay are still consistent with previous findings by others [18,24,25]. In addition, the method is very similar to the one used by Longo et al. [25], but they reported no standard deviations, and, therefore, comparison is impossible. Whether or not the high standard deviation is caused by a biological phenomenon or by technical difficulties is unknown and needs to be investigated further. We are currently preparing for analysis of the *ltxA* gene expression by Nanostrings technologies; Nanostrings technologies are based on hybridization probes and require no amplification of RNA, thus, the inherent variabilities in the PCR technique will be avoided.

In the present study, we characterized 20 Ghanaian *A. actinomycetemcomitans*, serotype b, according to the LtxA expression and production by three different quantitative methods. The LtxA expression and production of the Ghanaian *A. actinomycetemcomitans*, serotype b, was not fully related with previously published results on leukotoxicity of the strains [20]. Therefore, these findings indicate that the non-JP2 genotype strains of *A. actinomycetemcomitans*, serotype b, originating from Ghana and previously reported on as highly leukotoxic, may have enhanced leukotoxicity, but probably not at comparable levels to JP2 genotype strains. Furthermore, the results from the present study showed a reasonable relationship between the expression of *ltxA* and the amount of produced and secreted LtxA. Conclusively, it is possible that only using one method to describe the LtxA-related virulence potential of *A. actinomycetemcomitans* could result in incomplete or misleading conclusions. Therefore, it is recommendable to use more than one method, combining analysis of both the leukotoxicity and the LtxA expression and/or production, when characterizing the leukotoxic potential of *A. actinomycetemcomitans*.

## 4. Materials and Methods 

### 4.1. Bacterial Strains and Cell Lysis Assay

Table 1 shows the characteristics of 45 *A. actinomycetemcomitans* strains of serotype b, originally collected as subgingival plaque samples. The highly leukotoxic JP2 genotype of *A. actinomycetemcomitans*, characterized by a 530-bp deletion in the LtxA gene operon, belongs to serotype b, and is in particular linked to individuals of African descent [20,35,36]. Out of the 45 strains studied, 20 isolates from Ghanaian adolescents were previously analysed in a cell lysis assay based on the determination of lactate dehydrogenase (LDH) release from the monocytic leukemia cell line, THP-1 [20] as an expression of the leukotoxicity. Of these 20 *A. actinomycetemcomitans* strains of serotype b, 15 were non-JP2 genotype strains, and, among these strains, five were characterized as low leukotoxic, five as intermediate leukotoxic, and five as highly leukotoxic (0–30% LDH release = low leukotoxicity, 31–60% = average leukotoxicity, and 60% ≤ = high leukotoxicity, respectively) according to the study by Åberg and coworkers [20] (Table 1). The remaining five Ghanaian strains from that study belonged to the JP2 genotype of *A. actinomycetemcomitans* and were all highly leukotoxic towards THP-1 cells (Table 1).

The results obtained by the three quantitative assays being Western blotting, ELISA, and mRNA expression assay in the present study were compared with cell lysis assay results obtained in a previous study reported by Åberg and coworkers [20]. In addition to the 20 strains from Ghana, the bacterial collection in the present study was expanded with another 25 strains collected mainly from individuals originating from Africa or with African origin for comparison reasons. These 25 *A. actinomycetemcomitans* strains were 17 JP2 and five non-JP2 genotypes of *A. actinomycetemcomitans*, serotype b, previously characterized by Haubek et al. [36], and three non-JP2 genotype of *A. actinomycetemcomitans*, serotype b, from a collection of Moroccan isolates not previously reported on. The complete collection of 45 *A. actinomycetemcomitans* strains, serotype b, was analysed by ELISA and in an mRNA expression assay for comparison of the two different methods determining leukotoxic production and expression of *A. actinomycetemcomitans*, respectively (Table 1).

All strains were transformed into smooth variants by repeated subculture before being analysed by three different biochemical methods.

### 4.2. LtxA Isolation from the Cell Pellet and the Growth Supernatant

For isolation of LtxA, 50 ml volumes of pre-warmed TY×2 (Tryptone-yeast) medium were inoculated with a few colonies from a chocolate agar plate and incubated for 24 h in an atmosphere of 5% CO_2_ in air to an OD_600, 1 cm_ at approximately 0.3. Tubes were centrifuged at 3000× *g* for 20 min, and 1 mL of the supernatant was transferred to Eppendorf tubes, supplied with sodium azide to 3 mM, and stored at −20 °C for later quantification of LtxA by ELISA and Western blotting. The cell pellet was isolated and five ml of 10 mM phosphate-buffered saline (PBS, 0.3 M NaCl) pH 7.2, containing 3 mM sodium azide, was added to the bacterial pellet with the purpose of releasing the cell membrane-attached LtxA as described by Johansson and co-workers [10]. The tube was vortexed and rotated end-over-end for 30 min at room temperature. One ml of the suspension of the cell pellet in buffer was transferred to an Eppendorf tube and prepared for quantification of LtxA by Western blotting.

### 4.3. Western Blotting 

Western blotting was performed on the 20 Ghanaian *A. actinomycetemcomitans* (Table 1) for semi-quantitative comparison of the level of LtxA between the different strains and for semi-quantitative comparison of the level of LtxA in the cell pellet extract and the growth supernatant. Cell pellet extracts for SDS-page were prepared from suspensions of the bacteria in five ml 0.3 M NaCl (as described above) and subsequently diluted 10-fold with SDS-containing sample buffer in order to reflect bacteria-bound LtxA in the 50 mL culture volume. Supernatant samples were prepared as described above. The Western blotting was performed as described by Reinholdt and co-workers [14] using a polyclonal rabbit anti-LtxA antibody (Ab-LtxA) (unlabelled for coating, labelled for detection) raised against the C-terminal half (recombinant) of the LtxA molecule in collaboration with the DAKO laboratories (Glostrup, Denmark). Briefly, samples of supernatant and cell pellet extract were applied to a 7% Tris-acetat buffered gel (Nu-PAGE™, Invitrogen) in identical volumes in neighbouring lanes, providing visual semi-quantitative comparison of LtxA.

### 4.4. ELISA for Quantification of LtxA in Growth Supernatants

Quantification of the production of LtxA was performed by an ELISA as described by Reinholdt and co-workers [14]. Briefly, the LtxA isolated in the growth supernatant treated with sodium azide to 3 mM was used to quantify the LtxA production of the different strains. To detect LtxA by ELISA, polystyrene microplates (Nunc, Roskilde, Denmark) were coated overnight with Ab-LtxA in 10 mM phosphate-buffered saline, pH 7.4 (PBS). After washing and blocking the plate with washing solution (PBS containing 0.25 M NaCl and 0.15%Tween 20), test samples appropriately diluted in washing solution were incubated in wells for 2 h. Bound LtxA was detected by sequential incubations with biotinylated Ab-LtxA and alkaline phosphatase-conjugated streptavidine (DAKO). The assay was developed with a chromogenic substrate of p-nitrophenylenephosphate in diethanolamine buffer, pH 9.0, and plates were read at 405 nm by a Multiscan RC reader (Labsystems). The analysis was performed in duplicates, and the original strain HK921 (JP2) served as a reference. The results are the mean of the two runs and are given as a percentage of the results when testing *A. actinomycetemcomitans* strain HK921 (JP2).

### 4.5. mRNA Analysis

The purification of RNA, the synthesis of cDNA, and the performing of the real-time PCR for the determination of the expression of mRNA, coding for the production of LtxA in each strain, were performed as described by Søndergaard and co-workers with a few modifications [37]. Briefly, bacterial suspensions were made with smooth colonies in 1.5 ml Brain Heart Infusion (BHI) broth to an OD_600_ at approximately 0.4. The suspensions were centrifuged, and the cell pellets were isolated. The cell pellets were re-suspended in one ml of RNAprotect (Qiagen, by GmbH, Hilden, Germany), and the RNA was purified with Magna Pure Compact instrument using a MagnaPure Compact Nucleic Acid Isolation kit (large volume) (Roche Diagnostics GmbH, Mannheim, Germany). Residual DNA was degraded from the RNA sample using a Turbo DNA free kit (Ambion by ThermoFischer Scientific, Waltham, Massachusetts, USA) and 50% more DNase than recommended by the manufacturer in a Veriti 96-well Thermal Cycler (Applied Biosystems by ThermoFischer Scientific, Waltham, Massachusetts, USA) for 30 min at 37 °C, and 5 min at 95 °C. cDNA was prepared with TaqMan Reverse Transcription reagents (Life Technologies) in a Veriti 96-well Thermo Cycler for 10 min at 25 °C, 30 min at 48 °C and 5 min at 95 °C. The complete digestion of the genomic DNA in each RNA sample was confirmed by running a cDNA reaction without reverse transcriptase in the cDNA reaction mix, followed by PCR. The cDNA was mixed with primers, probes, and TaqMan Fast Advanced Master Mix (Life Technologies by ThermoFischer Scientific, Waltham, Massachusetts, USA) and quantitative PCR was run in triplicates in a LightCycler 480 (Roche, Germany). Relative gene expression of *ltxA* was quantified with LightCycler Relative Quantification software (Roche Applied Science) and normalized to the single-copy housekeeping genes *adk* and *pgi*. The assay was performed in triplicates, and the results are given as the mean of the three runs. Primers and probes are listed in Table 2.

### 4.6. Statistical Analysis

All the statistics were analyzed by the use of SciPy [38] that is an open source scientific tool for Python^®^ (Beaverton, USA). The statistical analysis were performed on log-transformed data in order to attain normal distributed data.

The difference between the group of JP2 and the group of non-JP2 genotype strains of *A. actinomycetemcomitans*, serotype b, determined for each method separately was analyzed by an unpaired sample t-test for parametric data.

A scatter plot for comparison of ELISA and the mRNA expression assay was performed as described by Ludbrook [33] with a few modifications. The relationship between the two methods was tested by use of a regression analysis. Because the ELISA quantifies the protein and the mRNA expression assay measures the mRNA coding for the protein, the two methods do not measure the exactly same quantity of the *A. actinomycetemcomitans* strains. Therefore, an ordinary least squares regression model was used, since the relationship between the two methods can be viewed as a calibration problem [33].

## Figures and Tables

**Table 1:** Characterization of the collection of *A. actinomycetemcomitans* strains, serotype b.

Bacterial Strains	Genotype	Origin	Leukotoxicity *
1 G	non-JP2	Ghana	Average
153 G	non-JP2	Ghana	Average
212 G	non-JP2	Ghana	Average
217 G	non-JP2	Ghana	Average
640 G	non-JP2	Ghana	Average
443 G	non-JP2	Ghana	High
486 G	non-JP2	Ghana	High
575 G	non-JP2	Ghana	High
605 G	non-JP2	Ghana	High
638 G	non-JP2	Ghana	High
369 G	non-JP2	Ghana	Low
467 G	non-JP2	Ghana	Low
493 G	non-JP2	Ghana	Low
708 G	non-JP2	Ghana	Low
744 G	non-JP2	Ghana	Low
331 M	non-JP2	Morocco	
394 M	non-JP2	Morocco	
416 M	non-JP2	Morocco	
HK1605	non-JP2	USA	
HK908	non-JP2	Porto-Brazil ^1^	
HK911	non-JP2	Holland	
HK912	non-JP2	Holland	
HK975 (Y4)	non-JP2	USA	
437 G	JP2	Ghana	High
488 G	JP2	Ghana	High
524 G	JP2	Ghana	High
654 G	JP2	Ghana	High
666 G	JP2	Ghana	High
HK1199	JP2	USA	
HK1507	JP2	Cape Verde Isands-Sweden ^2^	
HK1519	JP2	Cape Verde Islands-Sweden ^2^	
HK1547	JP2	USA	
HK1609	JP2	USA	
HK1615	JP2	USA	
HK1626	JP2	Tel Aviv-Switzerland ^3^	
HK1630	JP2	Algeria-Denmark ^4^	
HK1631	JP2	Morocco-Denmark ^5^	
HK1651	JP2	Ghana-Denmark ^6^	
HK1659	JP2	Morocco-Denmark ^5^	
HK1702	JP2	Brazil	
HK1707	JP2	Brazil	
HK1990	JP2	Portugal-Holland	
HK2000	JP2	Morocco	
HK2017	JP2	Tel Aviv	
HK921 (JP2)	JP2	USA	

* As defined by Höglund Åberg and coworkers (2014): 0–30% LDH release = low leukotoxicity, 31–60% = average leukotoxicity and 60% ≤ = high leukotoxicity. ^1^ Originating from Portugal, ^2^ Originating from Cape Verde Islands, ^3^ Originating from Tel Aviv, ^4^ Originating from Algeria, ^5^ Originating from Morocco, ^6^ Originating from Ghana.

**Table 2:** Primers and probes used in the mRNA expression analysis of *A. actinomycetemcomitans.*

Gene	Primer F (5’ → 3’)	Primer R (3’ → 5’)	Probe (5’ → 3’)
*pgi*	TAACCATGCACTTCGTGTCTAAC	CACAAGGGTGGTTTCCGGATT	TGGACGGCACGCACATTGCGGAAAC
*adk*	GATATGTTACGTTCCGCGATCA	GCACTAATTGACCGGCATCCA	AGCTGGCACCGAGTTAGGCAAACAAGC
*ltxA*	CTGTCGCAGGGTTAATTGCCT	GATCAAATTGTTTCGCAATACCTAG	TGTGGTCAGCTTGGCAATCAGCCCTTTG

## Supplementary Materials

The following are available online at https://www.mdpi.com/2076-0817/8/4/211/s1, Figure S1. A semi-quantitative determination of the leukotoxin expression by Western blotting of 20 Ghanaian *A. actinomycetemcomitans*, serotype b, both JP2 and non-JP2 genotypes. The cell membrane-attached LtxA isolated from the cell pellet (C), and the released LtxA into the growth supernatant (S). HK921 (JP2) is illustrated as a reference. The LtxA is given as the band with a size of 116 kDa.
