# Supplementary material for: Differential Cell Lysis Among Periodontal Strains of JP2 and Non-JP2 Genotype of Aggregatibacter actinomycetemcomitans Serotype B Is Not Reflected in Dissimilar Expression and Production of Leukotoxin
Source: Pathogens. 2019 Oct 30;8(4):211. doi: 10.3390/pathogens8040211 (PMC6963569; doi:10.3390/pathogens8040211)
Supplement: Supplementary file 1 [file pathogens-08-00211-s001.pdf]

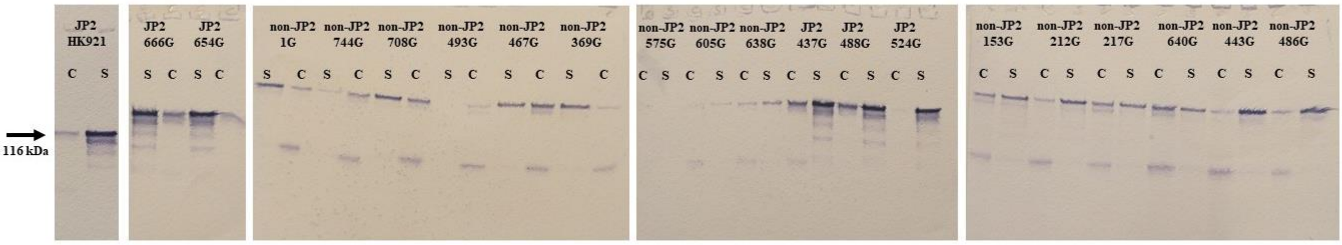

Figure 1. Western blotting of the twenty Ghanaian *A. actinomycetemcomitans*, serotype b, JP2 and non-JP2 genotypes showing a semi-quantitative determination of the leukotoxic production of the strains. The cell membrane-attached LtxA isolated from the cell pellet (C), and the released LtxA into the growth supernatant (S). HK921 (JP2) is illustrated as a reference. The LtxA is given as the band with a size of 116 kDa.
